# Retraction: Potential of antiviral peptide-based SARS-CoV-2 inactivators to combat COVID-19

**DOI:** 10.1371/journal.pone.0273489

**Published:** 2022-08-31

**Authors:** The PLOS ONE Editors

The *PLOS ONE* Editors retract this article [1] because it was identified as one of a series of submissions for which we have concerns about authorship, competing interests, and peer review. We regret that the issues were not addressed prior to the article’s publication.

ABG and MAA did not agree with the retraction. JL and ME responded but expressed neither agreement nor disagreement with the editorial decision. RMA and SMA either did not respond directly or could not be reached.

## References

[1] Gurung AB, Ali MA, Lee J, El-Zaidy M, Aljowaie RM, Almutairi SM (2022) Potential of antiviral peptide-based SARS-CoV-2 inactivators to combat COVID-19. PLoS ONE 17(6): e0268919. 10.1371/journal.pone.0268919 35657783 PMC9165783

